# Priming Dendritic Cells for Th2 Polarization: Lessons Learned from Helminths and Implications for Metabolic Disorders

**DOI:** 10.3389/fimmu.2014.00499

**Published:** 2014-10-20

**Authors:** Leonie Hussaarts, Maria Yazdanbakhsh, Bruno Guigas

**Affiliations:** ^1^Department of Parasitology, Leiden University Medical Center, Leiden, Netherlands; ^2^Department of Molecular Cell Biology, Leiden University Medical Center, Leiden, Netherlands

**Keywords:** antigen-presenting cells, dendritic cells, helminth, Th2 cells, obesity, metabolic diseases, insulin resistance, type 2 inflammation

## Abstract

Nearly one quarter of the world’s population is infected with helminth parasites. A common feature of helminth infections is the manifestation of a type 2 immune response, characterized by T helper 2 (Th2) cells that mediate anti-helminth immunity. In addition, recent literature describes a close association between type 2 immune responses and wound repair, suggesting that a Th2 response may concurrently mediate repair of parasite-induced damage. The molecular mechanisms that govern Th2 responses are poorly understood, although it is clear that dendritic cells (DCs), which are the most efficient antigen-presenting cells in the immune system, play a central role. Here, we review the molecular mechanisms by which DCs polarize Th2 cells, examining both helminth antigens and helminth-mediated tissue damage as Th2-inducing triggers. Finally, we discuss the implication of these findings in the context of metabolic disorders, as recent literature indicates that various aspects of the Th2-associated inflammatory response contribute to metabolic homeostasis.

## Introduction

Helminths are parasitic worms that infect one quarter of the world’s population. They classically evoke strong type 2 immune responses characterized by the induction of T helper 2 (Th2) cells, which secrete cytokines like IL-4, IL-5, and IL-13. These promote IgE production by B cells, and recruitment of eosinophils and alternatively activated macrophages. Together, these events control infection and/or mediate parasite expulsion through smooth muscle contraction and mucus production [reviewed in Ref. (1, 2)].

Helminths enter, migrate, and exit through their host, causing considerable tissue damage along the way. Therefore, it may not be surprising that recent literature has described a close association between type 2 immune responses and wound repair (3–6). In this context, a Th2-cell associated response would contribute to both wound repair and control of parasite infection, and seems beneficial over a type 1 response, which harbors a greater risk of inducing collateral tissue damage (1). In addition, various aspects of the type 2 immune response have been shown to contribute to metabolic homeostasis (7). Indeed, helminths were recently found to protect against diet-induced insulin resistance (8, 9), and a negative association exists between helminth infection and metabolic syndrome (10).

The mechanisms that initiate Th2 responses are not fully understood, even though it is clear that dendritic cells (DCs), the most efficient antigen-presenting cells (APCs) in the immune system, play a crucial role (11). Since helminths are the strongest natural inducers of type 2 immune responses, many advances in dissecting the mechanisms underlying Th2 polarization have been made using either models of helminth infection or helminth-derived products. In this Mini Review, we discuss recent advances in the field, examining both helminth antigens and helminth-mediated tissue damage as triggers for the initiation of a Th2 response. In addition, we discuss the potential implications of these findings in the context of metabolic disorders.

## Dendritic Cell Subsets Associated with Th2 Polarization

The importance of DCs in Th2 skewing is highlighted by studies showing that depletion of CD11c^+^ DCs interferes with the induction of a Th2 response to *Schistosoma mansoni* and *Heligmosomoides polygyrus* (12–14). Interestingly, it has become increasingly clear that distinct DC subsets induce different Th responses [reviewed in Ref. (11, 15)], and in the last few years, several studies analyzed the role of DC subsets in the initiation of Th2 responses to helminth infection. For example, two independent groups recently showed that the development of a Th2 response to *Nippostrongylus brasiliensis* depends on dermal CD301b^+^ DCs (16, 17). Specifically, depletion of CD301b^+^ DCs prior to infection reduces IL-4 production by CD4^+^ T cells, without affecting the percentage of T follicular helper (Tfh) cells or germinal center B cells (16). Mechanistically, Th2-inducing PDL2^+^CD301b^+^ DCs were shown to depend on DC-specific expression of the transcription factor interferon regulatory factor 4 (IRF4) (17). In line with these findings, CD11c^int^MHCII^hi^ dermal DCs expressing PDL2 and CD301b were also identified as a Th2-priming DC subset in *N. brasiliensis* infection (18). Of note, CD301b^+^ DCs alone are insufficient to generate a Th2 response *in vitro* (17) or *in vivo* (16), suggesting that additional requirements exist. For example, optimal localization of DCs within the lymph node may play a crucial role. In *H. polygyrus* infection, CXCR5-expressing CD11c^+^ DCs migrate to the lymph node and localize adjacent to B cell follicles (19). Depletion of CXCR5 or B cell-derived lymphotoxin alters the localization of the DCs and, as a consequence, impairs the development of Tfh and Th2 cells (19). In addition, it has been suggested that DCs require signals from basophils (20) and group 2 innate lymphoid cells (ILC2s) (21) to prime Th2 responses to allergens. Together, these studies suggest that specific DC subsets, as well as the microenvironment in which these subsets encounter CD4^+^ T cells, are important for Th2 development *in vivo*.

## Priming Dendritic Cells for Th2 Polarization

### Sensing helminth-derived antigens

Dendritic cells are equipped with pattern recognition receptors (PRRs) that recognize a wide array of pathogen-associated molecular patterns (PAMPs). The classical paradigm describes that triggering of PRRs, including the Toll-like receptors (TLRs), RIG-I-like receptors, NOD-like receptors, scavenger receptors, and C-type lectin receptors (CLRs), induces DC maturation and subsequent antigen-specific activation of Th cells (22).

While signaling through most TLRs induces Th1/Th17 responses (23), Th2-inducing helminth-derived molecules have also been described to interact with DCs through TLR2, 3, and 4 (24–27). Although the schistosome-related glycan LNFPIII, which contains Lewis X (Le^X^) trisaccharides, requires TLR4 for Th2 skewing (28), various studies suggest that TLRs are dispensable for Th2 polarization by helminth antigens. For example, bone marrow-derived DCs (BMDCs) from TLR2- and TLR4-knockout mice can still skew Th2 when pulsed with *S. mansoni* soluble egg antigens (SEA) (29), and the TLR adaptor protein MyD88 is not required for Th2 skewing by SEA-stimulated splenic DCs (30). Interestingly, human monocyte-derived dendritic cells (moDCs) stimulated with phosphatidylserine lipids from schistosomes induce IL-10-producing T cells through TLR2 (25). Therefore, helminth products may employ TLRs for the induction of regulatory responses, but it seems that other PRRs are required for the initiation of a Th2 response.

Indeed, CLRs that sense helminth glycans play an important role in Th2 skewing. For example, SEA is internalized by moDCs through DC-specific ICAM-3-grabbing non-integrin (DC-SIGN), macrophage galactose-type lectin (MGL), and mannose receptor (MR) (31), and binds to Dectin-2 on BMDCs (32). Binding of SEA to DC-SIGN was shown to depend on Le^X^ (33), and a recent study showed that blocking DC-SIGN-associated signaling inhibits Th2 skewing (34). Likewise, excretory/secretory products from the tapeworm *Taenia crassiceps* (TcES) bind MR and MGL on BMDCs (35), and the Th2-skewing capacity of TcES is glycan-dependent (36). Since SEA and ES mixtures contain many different glycoproteins, it is difficult to pinpoint the receptor and/or the mechanism responsible for Th2 polarization. Therefore, an important contribution to the field was made when omega-1, a small glycoprotein expressing Le^X^ residues (37), was identified as the major immunomodulatory component in SEA (38, 39). Generation of a glycosylation mutant revealed that omega-1 requires its glycans to condition moDCs for Th2 skewing, and to prime Th2 responses both *in vitro* and *in vivo*. Specifically, MR but not DC-SIGN, mediates recognition and internalization of omega-1 (40). In sum, these studies indicate that helminth-derived antigen preparations can bind a variety of PRRs, which may induce distinct intracellular events that promote Th2 polarization.

### Sensing epithelial alarmins

Modulation of DCs for Th2 priming can also take place in the absence of PRR signals, in response to epithelium-derived cytokine alarmins that are released with tissue damage (41). For example, stimulation of human myeloid DCs with thymic stromal lymphopoietin (TSLP) primes naïve T cells to produce IL-4, IL-5, IL-13, and tumor necrosis factor alpha (TNF-α) (42). However, the role of TSLP in helminth infection remains controversial. While TSLP receptor (TSLPR) knockout mice fail to mount a protective Th2 response to *Trichuris muris* (43, 44), they do develop a Th2 response during infection with *S. mansoni* (45), *H. polygyrus* or *N. brasiliensis* (44). Interestingly, basophils rather than DCs were recently described to act as TSLP-dependent APCs for Th2 skewing in *Trichinella spiralis* infection (46).

A second relevant alarmin is IL-33, as stimulation of BMDCs with this cytokine promotes Th2 development (47, 48). In line with these findings, IL-33 treatment improves Th2 cytokine production and expulsion of *T. muris* (49), and mice deficient for the IL-33 receptor T1/ST2 fail to develop a Th2 response following injection with *S. mansoni* eggs (50). Importantly, T1/ST2 is not only present on DCs but also on lymphocyte subsets including ILC2s, which were shown to mediate *N. brasiliensis* expulsion in an IL-33-dependent manner (51).

Lastly, IL-25 induces the production of type 2 cytokines by ILCs, and IL-25-knockout mice show delayed initiation of type 2 cytokine responses and *N. brasiliensis* expulsion (52). Although IL-25 has not been described to act directly on DCs, it was shown to enhance cytokine production by Th2 memory cells activated by TSLP-conditioned myeloid DCs (53). Thus, multiple alarmins are released by epithelial cells and may act in concert on various immune cell types, to mediate the induction of a Th2 response against helminths or their eggs.

### Intracellular mechanisms associated with Th2 polarization

#### Signaling-dependent mechanisms

Pattern recognition receptor-mediated signaling classically induces DC maturation via mitogen-activated protein kinases (MAPK) (54). However, in contrast to microbial ligands, helminth products often fail to induce classical signs of maturation and are well-known to downregulate TLR-mediated maturation (31, 38, 55–60). Indeed, unlike many TLR ligands, Th2-inducing compounds fail to phosphorylate p38 MAPK but instead promote phosphorylation of p42/p44 MAPK (ERK1/2) [reviewed in Ref. (61)]. ERK1/2 stabilizes c-Fos, and inhibiting either c-Fos or ERK1/2 enhances IL-12 production by moDCs (62), suggesting that activation of this pathway suppresses Th1-polarizing cytokines. Likewise, TSLP promotes ERK1/2 phosphorylation (63) and fails to induce IL-12 production by myeloid DCs (42, 64).

It was noted that the NF-κB signaling pathway also seems to be involved in Th2 polarization, as SEA- or LNFPIII-stimulated BMDCs from NF-κB1 knockout mice fail to prime a Th2 response (65, 66). Furthermore, it was recently demonstrated that Le^X^ residues, via DC-SIGN, activate LSP1 in moDCs, leading to nuclear accumulation of the atypical NF-κB family member Bcl3 and downregulation of IL-12 mRNA. These events also seem required for SEA-induced T cell polarization, since silencing either LSP1 or Bcl3 interferes with Th2 skewing (34). Similarly, the Th2-inducing capacity of TSLP was shown to involve activation of NF-κB and STAT5 (63, 67).

Finally, SEA can signal through spleen tyrosine kinase (Syk) downstream of Dectin-2, activating the Nlrp3 inflammasome and increasing TLR-triggered release of IL-1β by BMDCs. However, infection of various inflammasome-deficient mice with *S. mansoni* demonstrated that activation of this pathway does not seem to favor any particular Th response (32). Thus, helminth antigens can activate signaling, and certain members of the NF-κB and ERK pathways in particular seem to play a role in Th2 polarization.

#### Signaling-independent mechanisms

**Figure 1:**
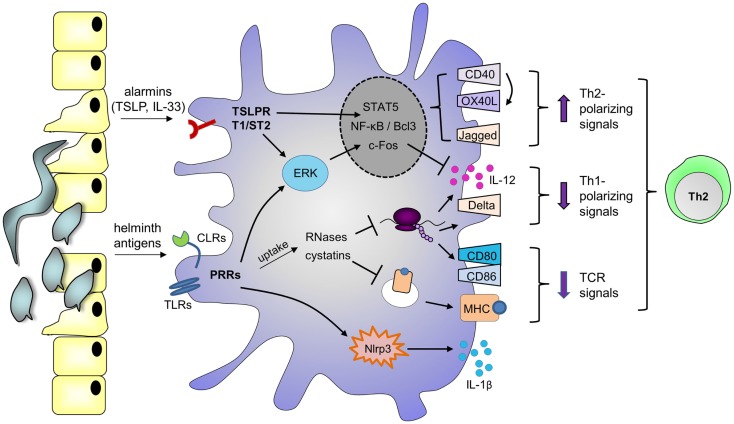
**Possible mechanisms by which helminth molecules modulate DCs for Th2 polarization**. Helminth antigens are recognized by DCs through ligation of pattern recognition receptors (PRRs), such as Toll-like receptors (TLRs) and C-type lectin receptors (CLRs). Depending on the antigen, binding promotes phosphorylation of ERK1/2, nuclear accumulation of NF-κB or Bcl3, and/or activation of the Nlrp3 inflammasome, which mediates IL-1β secretion. Phosphorylation of ERK1/2 stabilizes c-Fos, leading to downregulation of IL-12 expression. In addition, DCs can upregulate expression of Th2-associated CD40 and Jagged, which are under the control of NF-κB and ERK1/2, respectively (115, 116). Upon encounter of T cells expressing CD40L, signaling through CD40 promotes OX40L expression in an autocrine manner. Alternatively, PRRs may mediate uptake of antigens that interfere with antigen presentation on MHCs, such as cystatins, or RNases that inhibit protein synthesis, thereby suppressing the expression of costimulatory molecules like CD80 and CD86. These events affect T cell receptor (TCR) signaling. As a consequence of protein synthesis inhibition, RNases may also downregulate Th1-polarizing cytokines or molecules like IL-12 and Delta-4. In parallel, helminths or their eggs damage epithelium, and alarmins are released, such as thymic stromal lymphopoietin (TSLP) and IL-33, which bind the TSLP receptor (TSLPR) and T1/ST2, respectively. TSLP can also activate ERK1/2, STAT5, and NF-kB to promote CD40 and OX40L expression. Altogether, these events favor DC-mediated Th2 polarization.

In addition to signaling-dependent mechanisms, various helminth products harbor enzymatic activities that mediate Th2 skewing. For example, omega-1 depends on its RNase activity, which allows the molecule to cleave both ribosomal and messenger RNA, to downmodulate TLR-induced moDC maturation and IL-12 production, and to skew toward Th2 (40). Interestingly, various Th2-inducing allergens are also RNases (68, 69), as well as the endogenous eosinophil-derived neurotoxin that can amplify DC-mediated Th2 polarization (70). Together, these reports suggest that any RNase internalized by DCs may harbor Th2-priming capacities, through cleavage of ribosomal and/or messenger RNA. Similarly, a number of studies identified a role for cysteine protease inhibitors secreted by filarial nematodes (cystatins) in regulating host immune responses by interfering with antigen processing [reviewed in Ref. (71)]. Therefore, helminths may employ both signaling-dependent and independent mechanisms to condition DCs for Th2 skewing (Figure 1).

Of note, recent studies indicate that modulation of metabolic pathways within immune cells can regulate their function and, thereby, the outcome of the immune response (72). For example, BMDCs switch their core metabolism from mitochondrial oxidative phosphorylation to glycolysis upon TLR-ligation, and inhibition of this switch interferes with maturation, IL-12 expression, and the ability to induce CD4^+^ T cell proliferation (73, 74). Among the underlying mechanisms, the mammalian target of rapamycin (mTOR) was shown to control glycolytic metabolism (75, 76). Although we recently showed that mTOR is not involved in Th2 skewing by omega-1- or SEA-conditioned moDCs (77), the question whether helminths or their products affect glycolytic reprograming in DCs, and how this relates to Th2 polarization, constitutes an exciting new area of research.

## Primed DCs and Initiation of T Cell Polarization

A major difference between Th1 and Th2 development is that a Th1 response requires persistent production of Th1-polarizing cytokines, like IL-12, which are exclusively produced by APCs. By contrast, once primed DCs induce IL-4 production by a few activated Th cells, the Th2 response is self-sustained through autocrine production of IL-4 (78, 79). Therefore, in order to understand mechanisms of Th2 polarization, it is critical to identify the DC-associated polarizing signals that control early IL-4 production by activated T cells.

### Soluble factors and surface molecules

As discussed above, DCs stimulated with helminth molecules or TSLP fail to express IL-12. Moreover, injection of IL-12 can block the development of a Th2 response to *S. mansoni* eggs (80). These findings led to the so-called “default concept,” which states that Th2 differentiation spontaneously occurs in the absence of a Th1-priming signal like IL-12. However, mice lacking IL-12 do not develop a Th2 response to microbial pathogens (81), and blocking the mTOR pathway in LPS-stimulated moDCs skews a potent Th2 response without suppressing IL-12 secretion (77), suggesting that there are active signals involved in Th2 differentiation.

Such a signal may be provided by a soluble factor secreted by DCs, like RELMα, which was shown to promote IL-10 and IL-13 secretion by lymph node cells following adoptive transfer of SEA-stimulated BMDCs (82). However, supernatants from SEA-primed moDCs do not skew toward Th2 (83), and neither SEA- nor omega-1-stimulated BMDCs induce Th2 when separated from CD4^+^ T cells in transwells (39), indicating that an active polarizing signal in these studies is likely provided by surface molecules. Indeed, the Notch ligands Delta-4 and Jagged-2 have been linked to Th1 and Th2 polarization, respectively (84), and helminth antigens were shown to upregulate Jagged-2 on BMDCs (85, 86) and to suppress Delta-4 expression in moDCs (87). However, Jagged-2-deficient BMDCs can still skew Th2 when challenged with SEA (85, 86), suggesting that other molecules may be involved. For example, CD40 has been proposed to provide a polarizing signal, as its expression on SEA-stimulated BMDCs is required for the induction of a Th2 response (88), and mice lacking CD40 ligand suffer from impaired Th2 development during *S. mansoni* infection (89). Mechanistically, signaling through CD40 promotes OX40L expression, which is essential for optimal Th2 skewing by SEA-conditioned BMDCs (90) and moDCs (83), as well as TSLP-conditioned myeloid DCs (64). However, treatment with anti-OX40L does not significantly affect the Th2 response to *N. brasiliensis* infection (18), and it has been suggested that OX40L acts as a costimulatory molecule rather than a polarizing signal, since SEA-treated OX40L-knockout DCs induce Th2 cells, but fail to stimulate appropriate T cell expansion (90). Altogether, these studies suggest that there may not be one specific DC-associated molecule required for Th2 polarization, but rather a combination of signals that mediate both optimal T cell priming and expansion.

### Role for the T cell receptor

Early reports have described that the antigen dose can determine the outcome of Th differentiation, with a high dose generally favoring Th1 development (91–93). These findings were confirmed in a recent report, which also indicated that Th1-inducing adjuvants promote a higher Ca^2+^ flux [representing T cell receptor (TCR)-signaling strength], and induce larger synapse size, than Th2-promoting molecules (94). In addition, it has been suggested that T cells activated by Th2-inducing ligands are less proliferative, as priming of splenic DCs with SEA reduces the frequency of CD4^+^ T cells progressing through the cell cycle, and drug-induced arrest of cell cycle progression promotes Th2 polarization (30). Together, these observations suggest that helminth molecules may reduce TCR triggering, impairing T cell proliferation in favor of Th2 differentiation. Indeed, treatment of splenic DCs with SEA results in shorter T cell–DC interaction times and lower TCR signaling when compared to a Th1-inducing adjuvant (94). In addition, omega-1 reduces the capacity of BMDCs to form T cell–DC conjugates and diminishes the frequency of CD4^+^ T cells progressing through the cell cycle, possibly through modification of actin morphology (39). Mechanistically, interaction between T cells and DCs was shown to depend at least in part on the costimulatory molecule CD80 (94). As discussed above, helminth products fail to induce upregulation of costimulatory molecules, which may also explain why DCs treated with helminth molecules are less capable of forming stable interactions with T cells.

## Implications for Metabolic Disorders

The induction of a type 2 immune response has multiple functions. In the context of helminth infection, it mediates both parasite clearance and enhances wound healing. In addition, it has long been known that type 2 inflammatory responses contribute to the pathogenesis of allergy and asthma (95). Recently, however, it has become clear that multiple facets of the type 2 immune response are also involved in metabolic regulation (7). For just one example, IL-4 can regulate the balance between fatty acid and glucose oxidation in hepatocytes (96). Studying the molecular mechanisms that helminths employ to govern Th2 polarization may therefore open novel avenues for the treatment of metabolic disorders.

### Metabolic disorders and type 2 inflammation

A growing body of literature indicates that obesity is associated with chronic low-grade inflammation in metabolic organs. Enhanced infiltration of classically activated M1 macrophages, CD8^+^ T cells, and Th1 cells has been reported in both liver and adipose tissues (AT) (97). This represents a key etiological mechanism promoting tissue-specific insulin resistance and impairment in whole-body glucose homeostasis, which leads to an increased risk for type 2 diabetes and cardiovascular diseases. Interestingly, various reports have shown that Th2-inducing conditions, such as *N. brasiliensis* infection (8, 9), allergic inflammation (96), or SEA administration (98), improve insulin sensitivity and glucose tolerance in diet-induced obese mice. In addition, both *S. mansoni* infection (99) and SEA administration (100) reduce the development of atherosclerotic lesions in mice. Furthermore, adoptive transfer of CD4^+^ T cells (mostly via Th2 cells) and IL-4 treatment can protect against diet-induced insulin resistance (96, 101). Lastly, type 2-associated ILC2s (102, 103) and eosinophils (8) were shown to play a crucial role in maintenance of whole-body metabolic homeostasis by sustaining AT alternatively activated M2 macrophages. These findings are in line with epidemiological studies indicating that infection with helminths inversely correlates with metabolic syndrome (104, 105).

### Therapeutic manipulation of DCs for the treatment of metabolic disorders

**Figure 2:**
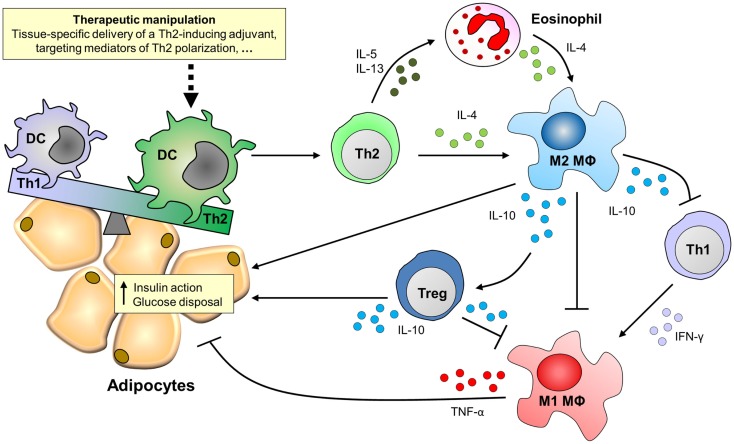
**Putative effects of targeting DCs for Th2 polarization on adipose tissue inflammation and insulin sensitivity**. Adipose tissues of obese patients and high-fat diet-fed mice are characterized by the accumulation of pro-inflammatory immune cells, like Th1 cells and M1 macrophages, which mediate tissue-specific insulin resistance through secretion of pro-inflammatory cytokines like IFN-γ and TNF-α. By contrast, M2 macrophages that secrete IL-10 protect against insulin resistance via multiple routes. For instance, IL-10 can act directly on adipocytes to potentiate insulin signaling, inhibit Th1 cells and M1 macrophages, and induce regulatory T cells (Tregs), thereby promoting adipose tissue insulin sensitivity and glucose disposal. The maintenance of M2 macrophages in adipose tissue depends on the presence of IL-4, which can be derived from Th2 cells or eosinophils. Novel treatment strategies may therefore focus on therapeutic manipulation of adipose tissue dendritic cells for Th2 polarization.

The ability of DCs to prime strong Th2 responses identifies these cells as an attractive target for therapeutic manipulation of the immune system in the context of metabolic disorders. DCs are widely studied as targets for development of vaccines and immunotherapies because of their capacity to regulate a wide array of T cell responses (106–108). It has been described that DCs accumulate in AT of obese patients and mice (109, 110), and therapeutic manipulation of DCs might also provide a new strategy for targeted treatment of metabolic disorders (Figure 2).

In terms of T cell priming, isolated AT CD11c^+^F4/80^low^ cells from obese mice were shown to preferentially induce Th17 responses (110), and AT CD11b^+^ APCs isolated from insulin-resistant mice promote Th1 polarization (111). However, since both AT macrophages and DCs can express CD11b, CD11c, and F4/80 (109), it is unclear which APC subset is responsible for these effects. In addition, targeting antigen to distinct DC subsets elicits distinct immune responses (112), and therefore, it remains to be determined whether AT DCs would be capable of polarizing Th2 responses *in situ*. These findings highlight the importance of studying AT-associated DC subsets, especially in humans, to identify appropriate subsets for therapeutic manipulation. Furthermore, it has been shown that antigen can efficiently be targeted to and processed by DCs *in vivo* using an antibody against CTL receptor DEC-205 (113, 114), a strategy which may be employed to target AT DCs. Toward this, a DC-restricted receptor on the appropriate subset needs to be identified. Lastly, directing Th2-inducing adjuvants to DCs requires single molecules that can easily be coupled to DC-specific ligands or antibodies. Therefore, proteins such as omega-1 hold promise (38, 39), since they provide a powerful tool to further dissect the molecular mechanisms underlying the induction of a DC-mediated Th2 response. In particular, the identification of the receptors and/or mediators involved in Th2 polarization will provide novel insights for the development of pharmaceutical agents that mimic helminth molecules in their modulation of DCs for Th2 skewing.

## Concluding Remarks

As this review illustrates, helminth molecules can interact with a variety of receptors, that either bind or internalize antigens to condition DCs for Th2 skewing through signaling-dependent and -independent mechanisms. *In vivo*, specific Th2-associated DC subsets are simultaneously exposed to polarizing signals from other immune cells or damaged epithelium. Depending on the helminth species and its migration through the tissue, it is likely that these signals act in concert to ensure robust Th2 polarization, although there seems to be some redundancy. It is now recognized that type 2 immune responses can also regulate energy metabolism, and studying how helminths generate Th2 responses will not only shed light on the mechanisms that promote control of parasite infection and wound healing but may also identify pathways that contribute to metabolic homeostasis.

## Conflict of Interest Statement

The authors declare that the research was conducted in the absence of any commercial or financial relationships that could be construed as a potential conflict of interest.
